# Co-Culture Approaches in Cartilage and Bone Tissue Regeneration

**DOI:** 10.3390/ijms26125711

**Published:** 2025-06-14

**Authors:** Iwona Deszcz, Julia Bar

**Affiliations:** Department of Immunopathology and Molecular Biology, Faculty of Pharmacy, Wroclaw Medical University, Borowska 211A, 50-556 Wroclaw, Poland

**Keywords:** co-culture, cartilage defects regeneration, bone defects repair, 2D co-culture models, 3D co-culture models

## Abstract

Cartilage and bone defects as well as osteoarthritis are prevalent worldwide, affecting individuals across all age groups, from young, active populations to older adults. The standard protocol in cartilage regeneration involves knee replacement surgery through the implantation of an endoprosthesis. Current clinical protocols involving cell-based therapies are associated with limitations, including the lack of functional cartilage-like tissue and dedifferentiation of chondrocyte, particularly during monoculture. Similarly, in bone regeneration, the “gold standard” is the use of bone auto- or allografts, which are associated with immunological rejection, inadequate vascularization, and limited osteogenesis. To overcome these limitations, various co-culture techniques have been introduced as promising strategies for cartilage and bone tissue regeneration. These systems aim to mimic native microenvironments by promoting interactions between chondrocytes and mesenchymal stromal cells (MSCs) in cartilage repair and between osteogenic and angiogenic cells in bone regeneration. This paper introduces different co-culture systems focusing on in vitro crosstalk between MSCs derived from various sources and other somatic cell populations in cartilage and bone regeneration.

## 1. Introduction

The articular cartilage in the knee may be injured during mechanical incidence or due to a degenerative disease, such as osteoarthritis (OA) [1]. Cartilage tissue has limited regenerative ability after injury due to a lack of blood and lymphatic vessels, nerves, and progenitor cells in the tissue structure [1,2]. A clinical standard of OA treatment is knee replacement by implanting a joint prosthesis onto the surface of the damaged cartilage tissue via surgical procedures [3]. Currently, two main approaches for cartilage defect repair, such as microfractures, based on bone marrow stimulation to activate endogenous mesenchymal stromal cells (MSCs) and autologous chondrocyte implantation (ACI), have been used [3,4]. However, the regenerative outcomes of these approaches remain limited and are considerably less effective compared with total joint replacement. Furthermore, current clinical therapies utilizing chondrocytes, MSCs, or progenitor cells are unable to fully restore the native biomechanical properties of articular cartilage [3,5,6,7]. MSCs are able to differentiate into chondrocytes, but the hypertrophic differentiation of in vitro MSC-derived chondrocytes induces maintained instability in cartilage [3]. Regarding chondrocytes, the predominant issue concerns the prevention of chondrocyte dedifferentiation during monolayer cultivation prior to ACI [5].

In parallel with cartilage repair, the field of bone regeneration has emerged as a major area of interest in regenerative medicine, particularly due to the clinical challenges associated with traumatic injuries, tumor resections, and degenerative diseases. Bone is dynamic tissue subjected to continuous remodeling throughout its lifetime in order to preserve its structure and function [8]. The loss of bone tissue caused by different defects or orthopedic diseases results in patient morbidity and remains a challenge in orthopedics and maxillofacial surgery [8,9]. The standard clinical treatment of damaged bone is based on allografts or synthetic bone reconstruction [10]. The main issue in bone reconstruction is inducing vascularized bone grafts, because bone tissue is a highly vascularized tissue containing numerous vessels and capillaries directly participating in the osteogenic generation of new bone [11]. Despite the promising clinical outcomes in bone augmentation, new clinical approaches to improved new tissue formation have been explored [11]. A promising alternative therapy is based on stem cells and tissue engineering. Several studies have concentrated on enhancing bone regeneration using MSCs [11,12,13]. According to published data, 15 clinical trials have investigated the use of MSCs to treat bone defects (clinicaltrials.gov (accessed on 17 February 2025)). During bone defect healing, MSCs are recruited through newly formed blood vessels, after which they differentiate into chondrocytes or bone-forming osteoblasts, ultimately leading to bone formation via endochondral or intramembranous ossification [14]. It was reported that the effectiveness of bone regeneration strongly depends on MSC differentiation into osteoblasts, which secrete the osteoid matrix that will be mineralized [15]. However, bone remodeling involves both osteoblasts and osteoclasts, and the balance between these cells plays a crucial role in bone matrix formation [12]. Similar to cartilage repair, several obstacles have been pointed out in the application of MSCs for bone regeneration, such as the homing of MSCs in damaged bone, short survival time after transplantation, optimal doses, and low osteogenic potential of MSCs [11].

It was pointed out that the administration of a single type of cell population, such as MSCs, progenitor stem cells, or chondrocytes, does not guarantee full recovery of cartilage or bone defects [3,5,11]. To overcome these limitations and maintain a stable phenotype typical for articular cartilage, alternative approaches using co-culture techniques have been developed to mimic complex and dynamic cellular interactions in native cartilage tissues and facilitate changes in cellular phenotypes during chondrogenesis [3,5]. Freshly isolated chondrocytes combined with an extracellular matrix (ECM) have been used to induce cartilage repair [16]. Studies have shown that co-cultures of chondrocytes with MSCs may enhance the chondrogenic capacity of MSCs and have high potential to induce cartilage regeneration [4,17,18]. On the other hand, bone regeneration introduces additional complexities, including hypoxia and nutrient deprivation, both of which contribute to substantial MSC death post-implantation. In this context, co-culture approaches involving cells with both angiogenic and osteogenic potential offer significant benefits. These include reduced apoptosis of MSCs and increased proliferation and differentiation potential of MSCs [19]. The main advantage of co-culture in bone regeneration lies in its ability to mimic the native bone microenvironment through the presence and interaction of both angiogenic and osteogenic cells, thereby facilitating effective and accelerated bone repair [9]. This regenerative synergy is mediated through paracrine signaling mechanisms and cell-to-cell contact between endothelial cells (ECs) and MSCs. ECs secrete pro-survival and pro-osteogenic factors, which enhance MSC viability, migration, and osteogenic differentiation. In turn, MSCs produce ECM components and osteoinductive cytokines that promote EC stabilization and neovascularization [20].

This paper introduces different co-culture systems used in cartilage and bone regeneration. It also reports on the in vitro crosstalk between frequently used MSCs from various sources and other somatic cell populations in the repair process of both tissues.

## 2. Various Treatments Used for Cartilage and Bone Lesions

### 2.1. Autologous Chondrocyte Implantation

Recently, ACI has become a standard clinical practice in some countries, as it helps reduce the side effects associated with cartilage tissue damage and may temporarily restore the flexibility and mechanical function of cartilage tissue and delay joint replacement surgery [7,17,21,22,23,24]. Repairing cartilage defects using autologous chondrocytes has the potential to regenerate cartilage, because these cells are responsible for the production of a cartilage matrix and support the ECM by releasing substances that make cartilage flexible, such as proteoglycans (aggrecan and collagen type II (COL2)) [25]. Unfortunately, the main challenges in using chondrocytes in cell-based therapy are not only obtaining a high number of these cells but also maintaining their phenotype and functionality. Chondrocytes have an extremely low in vivo and in vitro capacity for proliferation, and in vitro expansion of chondrocytes results in dedifferentiation. Dedifferentiated chondrocytes display altered morphology and phenotype features and reduced specialized function, such as the production of ECMs [18,26]. Another obstacle is that cartilage biopsy requires healthy cartilage, which may not always be available, especially in patients with extensive joint damage and advanced OA. Moreover, ACI is an expensive procedure. Firstly, in vitro culture takes several weeks to obtain a high number of cells. Secondly, it is a two-stage technique, in which the first surgery is used to obtain and isolate chondrocytes from the patient’s cartilage and the second surgery is used to implant the cultured cells into the defect site. Additionally, ACI is less effective for larger defects or in cases of widespread degeneration, such as OA, and should be performed soon after a cartilage injury as a primary intervention to enhance the tissue regeneration capacity [27]. These limitations have driven ongoing studies into alternative cartilage repair techniques.

### 2.2. Stem Cell-Based Therapies in Cartilage and Bone Repair

**Figure 1:**
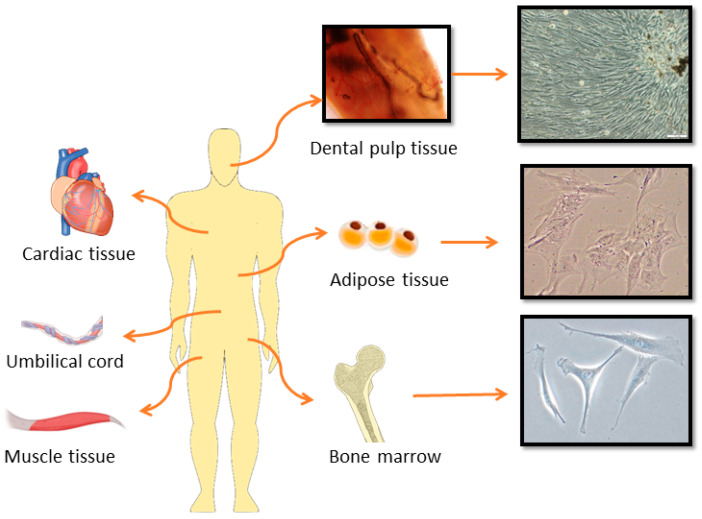
Mesenchymal stromal cells isolated from different tissues used in cartilage and bone regeneration (microphotographs present original authors’ unpublished data; magnification ×200, ×400, ×400 respectively).

Currently, MSCs are the most frequently utilized cells in cell therapies for various pathological and regenerative tissue conditions due to their proliferative potential, differentiation capabilities, immunomodulatory properties, and ease of acquisition [28]. MSCs are adult, multipotent stem cells that can be derived from a variety of tissues, including the bone marrow, adipose tissue, umbilical cord, dental tissues, placenta, and skeletal muscles (Figure 1) [29].

Regardless of the source of isolation, MSCs must follow the specific criteria set forth by the Mesenchymal and Tissue Stem Cell Committee of the International Society for Cellular Therapy (ISCT) [30]. The recommendations are as follows: expression of surface molecules such as CD44 (cluster of differentiation), CD73, CD90, and CD105 and negative expression of CD14 or CD11b, CD79a or CD19, CD34, CD45, and HLA-DR (human leucocyte antigen DR isotype) marker; in vitro and in vivo capabilities for self-renewal and differentiation; secretion of trophic factors; immunomodulation; and support processes such as angiogenesis. Furthermore, validating functional matrix activity requires the use of several analytical methods, including quantitative RNA expression profiling of target genes, flow cytometry analysis of surface antigens, and protein-level characterization of the MSC secretome [30]. Most importantly, the chondrogenic potential of MSCs, along with their anti-inflammatory, anti-apoptotic, anti-oxidative, and immunomodulatory properties, enables them to treat cartilage defects, which affect not only elderly individuals but also young people and especially athletes, who overstrain their joints and suffer injuries [31]. Inga Urlić and Alan Ivković [32] reviewed strategies for using various cells, including MSCs, from different sources in the treatment of cartilage defects and their potential clinical applications, highlighting promising results in the field of regeneration for patients with joint diseases. Similar to cartilage regeneration, adult stem cells isolated from various tissues (Figure 1) are also promising candidates for bone tissue regeneration due to their ability to stimulate osteogenesis and angiogenesis [15,29]. Moreover, according to published data, successful regeneration of bone tissue defects requires stem cells with high osteogenic potency [33]. Table 1 summarizes the chondrogenic and osteogenic potential of MSCs derived from different sources frequently used for cartilage and bone regeneration.

#### 2.2.1. Cartilage Tissue Repair

To date, MSCs from different sources have been used for cartilage regeneration. The most commonly used stem cells in cartilage tissue regeneration are the patient’s native bone marrow-derived MSCs (BMSCs) and adipose-derived MSCs (ADMSCs) [38,39]. In clinical settings, the aforementioned MSCs can be administered intra-articularly to regenerate tissue, alleviate inflammation (by secreting interleukin (IL)-10 and transforming growth factor β (TGF-β)), reduce pain in patients with moderate OA, and stimulate the quiescent resident MSCs to enhance therapeutic effects [40]. It was found that BMSCs showed high chondrogenic potential and effectively promoted the regeneration of articular cartilage, and they could be used in both autologous and allogeneic applications, but their isolation is an invasive clinical procedure, which may lead to complications [41,42]. ADMSCs are also widely used in cartilage regeneration; however, studies have shown that they possess lower chondrogenic potential and produce less ECMs compared with BMSCs [43,44]. The collection of ADMSCs not only involves an invasive procedure but also results in a low number of stem cells in the harvested material [45]. On the other hand, human umbilical cord MSCs (hUC-MSCs) offer several benefits, which are shown in Table 1. Studies have demonstrated that hUC-MSC implantation is safe and effective for cartilage repair in older patients with knee osteoarthritis [35,46].

Despite some limitations, stem cell therapies have shown promising results in cartilage regeneration due to their differentiation potential and production of a variety of ECM molecules, such as collagens, proteoglycans, glycosaminoglycans, and fibronectin [47]. However, some clinical trials indicate that in OA, intra-articular MSC injections lead to short-term improvements in various outcome measures, but their long-term effectiveness tends to decline over time [48]. This may be caused either by MSC migration into the bloodstream or cell death at the defect site. Nevertheless, a study by Shang et al. [49] showed that the regenerative potential of MSCs may be adversely affected by the disease environment.

#### 2.2.2. Bone Tissue Repair

Several studies have investigated the usefulness of MSCs in bone tissue regeneration by assessing their osteogenic differentiation capacity and regenerative potential [15,33,50]. In addition to their differentiation capacity, MSCs secrete bioactive molecules that regulate immune responses and stimulate the formation of new blood vessels, thereby creating a regenerative microenvironment that is conducive to effective bone repair. Among various types of stem cells, most clinical and preclinical studies have investigated the utility of BMSCs and ADMSCs [51]. BMSCs are most commonly used due to their high osteogenic capacity and their ability to differentiate into osteoblasts and chondrocytes, which are essential for early formation of the bone matrix and cartilage during healing [52]. Moreover, BMSCs’ secret molecules enhance osteoblast proliferation (fibroblast growth factor (FGF)-2, bone morphogenetic protein (BMP)-2, BMP-4, TGF-β, and insulin-like growth factor 1 (IGF-1)), angiogenesis (vascular endothelial growth factor (VEGF)), and immune modulation, thereby creating a microenvironment conducive to bone repair [53]. ADMSCs have also demonstrated promising outcomes in clinical studies, promoting bone regeneration not only through osteogenic differentiation but also via secreting a range of pro-regenerative factors, including VEGF, bone morphogenetic proteins (BMP-2 and BMP-4), IGF-1, IL-8, hepatocyte growth factor (HGF), macrophage colony-stimulating factor (M-CSF), and receptor activator of nuclear factor kappa-B ligand (RANKL) [34,37]. However, comparative data suggest that ADMSCs exhibit lower osteogenic potential than BMSCs but possess a greater ability to promote angiogenesis [34,36,54]. Other authors showed that ADMSCs, compared with human dental pulp stem cells (hDPSCs), demonstrate higher osteogenic potential, higher expression of osteoblast marker genes, and greater mineral deposition [37]. On the other hand, hDPSCs promote better angiogenesis than other MSCs, because they secrete a higher level of VEGF [37].

**Figure 2:**
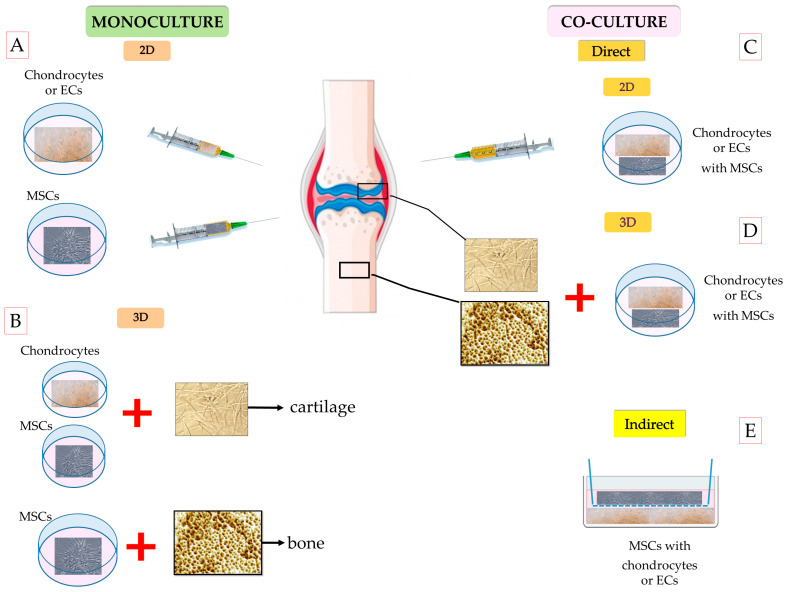
Regeneration of cartilage and bone tissue using mono- or co-culture models with MSCs and chondrocytes or endothelial cells (ECs): (**A**) cell-based therapy, using chondrocytes or MSCs in cartilage repair and MSCs or ECs in bone repair as monoculture; (**B**) 3D model (membrane), with chondrocytes or MSCs in cartilage and MSCs in bone repair; (**C**) direct co-culture of chondrocytes with MSCs in cartilage regeneration and co-culture of MSCs and ECs in bone regeneration; (**D**) direct 3D co-culture system for cartilage (MSCs + chondrocytes) and bone (MSCs + ECs) repair; and (**E**) indirect co-culture systems for cartilage and bone repair. (**A**–**C**, magnification ×200).

Despite extensive research, stem cell-based therapies for bone regeneration have not yet been approved for standard clinical use and remain limited to clinical trials. In contrast to cartilage repair, bone regeneration is more complex, as it requires the coupling of both angiogenesis and osteogenesis. A recent meta-analysis by Cui et al. [55] demonstrated that stem cell-based therapies significantly improved bone healing outcomes but primarily in the short term. In light of these findings, further research is needed to standardize treatment protocols, assess long-term safety and efficacy, and identify the most suitable stem cell sources and delivery methods for clinical application. It is worth emphasizing that during cartilage and bone regeneration, MSCs may undergo senescence due to aging, oxidative stress, and inflammatory conditions. These processes decrease the effectiveness of therapy due to reduced secretion of pro-inflammatory cytokines and the proliferation and differentiation potential of MSCs [15,56]. To solve these obstacles, researchers recommend stimulating MSCs with other types of cells in co-culture systems [57,58]. Figure 2A–E presents frequently used monoculture and co-culture models in cartilage and bone tissue repair.

## 3. Co-Culture Systems for Cartilage and Bone Regeneration

Co-culture systems, which apply more than two different types of cells in one culture dish, have been used to study cell–cell communication mechanisms in native tissue and establish tissue-engineered grafts for medical research and clinical application [9]. It was found that cell–cell interactions may lead to better comprehension of some phenomena occurring in vivo and improve culture efficiency [9]. Therefore, considerable effort has been expended into developing co-culture models for specific tissues, including the liver, pancreas, retina, skin, tendon, cartilage, vessels, nerve, and bone [26,59]. The main advantage of co-culture over monoculture is that it facilitates interactions among the different cell types via intercellular signal transmission through junctions, exosomes, and paracrine activity, as is observed in native hard and soft tissues [9]. However, there are difficulties involved with co-culture systems, such as proper selection of the parameters for the co-existence of two or more different cell types, namely the cell ratio, shared medium, time points, imaging, and cell functions. The co-culture model can be set up both in a two-dimensional (2D) and three-dimensional (3D) arrangement, with or without direct physical contact among the different cell types (Figure 2C–E).

### 3.1. Co-Culture in Cartilage Regeneration

#### 3.1.1. 2D Co-Culture Model for Cartilage Tissue Regeneration

Cartilage tissue engineering is one of the areas that has been studied most extensively to develop new possibilities in cartilage repair [60]. One main issue is the production of ECM by chondrocytes containing COL2 and aggrecan [61]. Therefore, many researchers focused their attention on co-culture systems that interplay between different cell types to enhance factor secretion for the activation of cell signaling pathways, leading to the stimulation of cartilage formation [11,17,46,62,63,64]. As shown in Figure 2, different co-culture models have been tested in cartilage repair.

It is worth underlining that BMSCs are the most frequently chosen MSCs for co-culture with primary chondrocytes. However, ADMSCs, human Wharton’s jelly MSCs (hWJ-MSCs), synovial mesenchymal stromal cells (SMSCs), human dental stem cells (hDMSCs), and hUC-MSCs have been also tested in this culture model [29,64,65,66,67]. Some reports indicate that MSCs isolated from different sources share common features; however, there are significant differences in their biological behavior [29,64,65,66,67,68]. Clinical trials have demonstrated that hWJ-MSCs can effectively alleviate OA symptoms and reduce pain, highlighting their potential for therapeutic applications in degenerative joint disorders [67]. Nevertheless, there are studies suggesting that SMSCs, compared with other MSCs, have the best chondrogenic potential confirmed by cartilage repair, resulting in clinical and histological outcomes [66,68]. In co-culture with chondrocytes, the upregulation of matrix formation markers is induced compared with SMSC monoculture [62], while hDPSCs and stem cells from human exfoliated deciduous teeth (hSHED), due to their easy accessibility, multipotency, immunomodulatory capabilities, and high proliferative ability, are valuable candidates for therapeutic applications [69]. These cells showed effective chondrogenic differentiation, especially hSHED [70]. Currently, most studies investigating hDMSCs in cartilage repair are still in experimental phases or based on animal models [69,71,72,73]. However, one study has demonstrated that hDPSCs are a valuable cell source for the regeneration of fibrocartilage in joints [74]. Despite glycosaminoglycan, aggrecan, and limited COL2 expression, these cells produced a collagen-rich ECM predominantly composed of COL1 after 21 days of differentiation in a pellet culture model [74]. These findings highlight that not all types of MSCs possess strong chondrogenic differentiation potential in hyaline cartilage [75]. Therefore, selecting the appropriate cell source and optimizing the culture environment are critical factors for successful cartilage regeneration. The first-in-human study revealed that allogeneic MSCs in combination with chondrons stimulate tissue regeneration through paracrine mechanisms and cellular communication, proving to be a safe cell source to enhance or facilitate tissue regeneration in clinical settings [76]. On the other hand, Marchan et al. [17] showed that 2D co-culture of chondrons and BMSCs has a negative impact on hyaline cartilage formation, because BMSCs induced the dedifferentiation, rather than promotion, of chondrogenic genes and ECM production by chondrons. However, another study showed that chondrocytes stimulated the chondrogenic differentiation of hUC-MSCs in direct co-culture by reducing fibrocartilage formation during tissue regeneration [65]. Researchers observed a reduction in COL1’s presence in regenerated cartilage, indicating a shift toward a more hyaline-like cartilage phenotype [65]. In contrast, ADMSCs in co-culture with chondrocytes were found to be less effective than BMSCs at stimulating chondrogenic markers [77,78].

**Figure 3:**
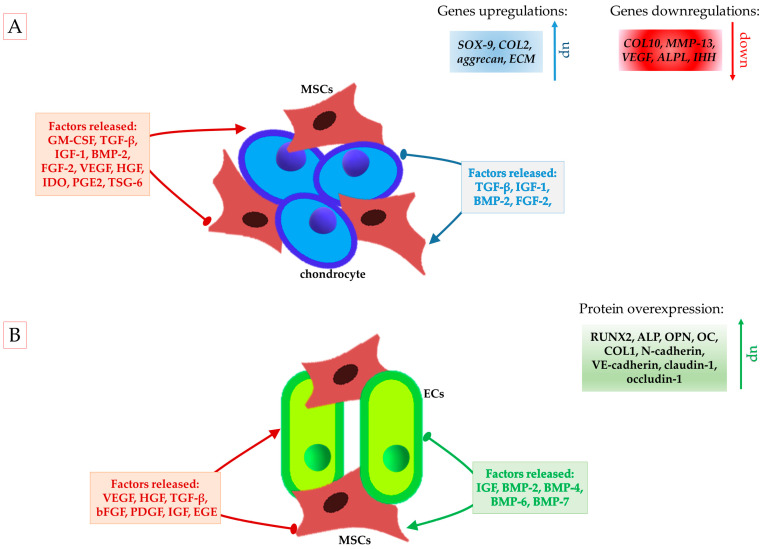
Crosslink between various types of cells in cartilage and bone regeneration. (**A**) Proteins released by direct co-culture of chondrocytes and MSCs in cartilage regeneration. (**B**) Active factors released by MSCs and ECs in direct co-culture model, promoting angiogenesis and enhancing osteogenic potential of MSCs during bone regeneration.

The implementation of direct co-culture techniques may present a viable solution to the issue of phenotype changes in chondrocytes. This approach is advantageous over monoculture methods, primarily because it requires a lower quantity of chondrocytes. Additionally, it reduces the time required for cell proliferation, thereby shortening the process of increasing the cell number in monolayer cultures. Co-culture may potentially eliminate the need for culture expansion using freshly isolated primary chondrocytes [79]. This method not only saves valuable time and resources but also potentially enhances the quality of chondrocyte cultivation, leading to more consistent and reliable results in tissue engineering applications. Cell–cell contact, combined with trophic factors, is crucial for cartilage regeneration, as it enhances the proliferation and expression of chondrogenic genes (such as *SOX-9*, *COL2*, and *aggrecan*) and the production of ECM by chondrocytes [80]. Moreover, it leads to the production of anti-inflammatory factors, including TGF-β, tumor necrosis factor-stimulated gene-6 (TSG-6), HGF, nitric oxide (NO), heme oxygenase-1 (HO-1), indoleamine 2,3-dioxygenase (IDO), prostaglandin E2 (PGE2), and HLA-G, which influence the secretion of pro-inflammatory cytokines, such as IL-1β, IL-8, and IL-6 [81]. Inflammation, which involves the production of matrix metalloproteinases such as MMP-1, MMP-3, and MMP-13, negatively affects the cartilage matrix quality and quantity [3]. Moreover, MSCs protect cells from apoptosis through the secretion of VEGF, HGF, IGF-1, TGF-β, and GM-CSF [81]. It was revealed that primary chondrocytes may influence MSC chondrogenesis by secreting a variety of protein molecules, such as TGF-β, IGF-1, BMP-2, and FGF-2 [82]. This cooperation also affects the stabilization of the phenotype of MSCs (chondro-induced) by reducing hypertrophic chondrocytes. A prominent feature of hypertrophic chondrocytes is a significant level of collagen type X (COL10), rather than COL2, and alkaline phosphatase (ALP), which facilitates endochondral bone formation [83,84]. Hypertrophic chondrocytes are undesirable during cartilage regeneration, because their presence initiates a process in which the cartilage undergoes ossification. This leads to the invasion of blood vessels and osteogenic cells, ultimately resulting in the replacement of cartilage with bone tissue [85]. A growing number of studies proved that the co-culture conditions influence the phenotype of chondrocytes by producing higher levels of ECM and proteins, such as COL2, SOX9, and aggrecan, and the phenotype of MSCs, reducing their hypertrophic phenotype after chondrogenic differentiation [3,86,87]. According to published data, cartilage formation through MSC and chondrocyte co-culture is enhanced by communication and interactions between cells and secreted factors. Therefore, direct co-culture seems to be better than an indirect model [3,20,88]. As presented in Figure 3A, the direct co-culture of MSCs and chondrocytes increases the secretion of paracrine factors which facilitate cartilage repair.

Moreover, for cartilage tissue engineering, the quantity of cells utilized for injection is a critical factor that can significantly influence the success of the regenerative process. A meta-analysis of clinical trial results showed that in knee osteoarthritis, significant improvement in pain and functional outcomes was observed in patients who received monocultures of MSCs at a dose of 50–100 million [89]. In the co-culture system, it is also important to use the optimal ratio of chondrocytes and MSCs to obtain optimized tissue-engineered cartilage with enhanced mechanical properties and reduced ossification risk. According to research, the best cell ratio is more chondrocytes than MSCs or an equal number of both [17,80]. This ratio supports chondrocytes in maintaining their phenotype better and exhibiting increased expression of cartilage-specific markers in ECM production. It also affects MSCs’ differentiation into chondrocytes [17,64]. Additionally, MSCs have a positive effect on chondrocyte proliferation. Research indicates that monoculture and indirect culture do not show an increasing number of chondrocytes during culturing [90].

#### 3.1.2. Tissue Engineering Strategies Using 3D Co-Culture Models for Articular Cartilage Defects

The 2D direct co-culture of MSCs and chondrocytes offers a promising avenue for future clinical applications in cartilage repair. However, challenges remain, particularly regarding the stability of the cells at the injection site, and the formation of proper hyaline cartilage has not yet been achieved. Furthermore, the approach is currently limited by the inability to effectively address large cartilage tissue defects. To solve this issue, cells can be combined with membranes or biomaterials that facilitate tissue repair (Figure 2B,D). Moreover, 3D culture systems, such as scaffold-free in the form of pellets or spheroids and scaffold-based (membranes), may help obtain a physiologically relevant environment for hyaline cartilage formation and reduce chondrocyte hypertrophy [91].

The “gold standard” culture model for the induction of chondrogenic differentiation is pellet culture [92,93,94]. Many studies have demonstrated the positive effects of co-culture with pellets on chondrocyte hypertrophy and the enhancement of chondrogenesis [88,90,94]. It was observed that at the same time as the gene expression of *COL10*, *MMP-13*, *VEGF*, *ALPL*, and *hypertrophic transcription factor IHH* decreased, chondrogenic gene expression (*COL2*, *aggrecan*, and *SOX9*) increased compered with the monoculture of chondrocytes [95]. The authors showed that pellet co-culture in an in vivo OA model enhanced the creation of functional cartilage-like tissues [79,90,93,95,96]. Moreover, factors regulating matrix remodeling, such as TIMP-1 and TIMP-2, as well as cell proliferation and the synthesis of cartilage-specific proteins, such as TGF-β3, FGF-4, FGF-6, G-CSF, and GM-CSF, were observed [90]. Additionally, key regulators of MSC commitment and differentiation, such as TGF-β, RANTES, IL-2, and IL-12, as well as leukemia inhibitory factor (LIF) were significantly upregulated in MSC and chondrocyte co-culture [90]. In vivo models confirmed that mixed co-culture of chondrocytes and MSCs can differentiate into functional cartilage-like tissues [95]. However, despite the above advantages, using cell pellets has some limitations. Firstly, there is a need for a high cell number and a lack of structural support. Secondly, pellets may only be effective for filling small cartilage defects and are not suitable for repairing larger defects with clinically relevant dimensions [97]. To improve the effectiveness of cartilage regeneration, a spheroid co-culture system has been analyzed, because it offers several advantages over pellet culture. First of all, it requires significantly fewer cells and is easier to handle [98]. Furthermore, the use of spheroids in MSC transplantation is well established in tissue engineering, with evidence showing that they can improve the therapeutic potential of MSC post-transplantation [99]. Moreover, the spheroid culture system enhances MSCs’ properties, including their anti-inflammatory and angiogenic capabilities, increases stemness, facilitates differentiation into various cell lineages, and improves cell survival after transplantation, as well as paracrine factor secretion (angiogenin (ANG), angiopoietin 2 (ANGPT-2), IL-11, bFGF, FGF-2, VEGF, and HGF) [100].

Based on published data on cartilage regeneration, it may be assumed that the use of scaffolds as a platform for cells is the dominant approach in cartilage therapy [88]. Up to now, different natural or synthetic biomaterials combined with chondrocytes or MSCs have been investigated in preclinical and clinical studies [101,102,103,104]. Monoculture on scaffolds is generally accepted for cartilage repair, but it was found that it is not an efficient biomaterial for cartilage tissue engineering because of the lack of chondrogenic markers [63]. To date, different 3D environments for co-culture, such as hydrogels and membranes, have been analyzed that improve cartilage formation [63,91,102,105]. The standard 3D co-culture model is shown in Figure 2D. It is worth highlighting that in our previous study, we used a Hyaff-11 hyaluronic acid membrane in a direct 3D co-culture model of chondrocytes with BMSCs, but other groups mostly used hydrogels as a 3D co-culture system [63]. In this case, independent from the spatial arrangement, mechanical properties, and surface characteristics of the scaffolds, it acted similarly by increasing the expression of cartilage markers and deposition of cartilaginous ECM compared with 2D monoculture [88,106,107]. As with standard 3D culture, MSCs and chondrocytes in 3D co-culture respond to local microenvironmental stimuli, resembling in vivo conditions. This interaction supports the maintenance of the differentiated phenotype of chondrocytes and the chondrogenic differentiation of MSCs, leading to the promotion of more functional cartilage tissue development in engineered constructs [72]. The 3D structure allows cells to form natural cell-cell junctions, improving intercellular communication through gap junctions, which facilitates the transmission of ions and signaling molecules that regulate chondrogenic differentiation [72]. Additionally, cell–ECM interactions occur, which affect cell behavior, including cell differentiation, proliferation, and gene expression [108]. It has been shown that natural biomaterials, due to the biological cues between the cells and the scaffold, provide enhanced chondrogenesis through hyaline cartilage rather than synthetic biomaterials, such as polycaprolactone and polyglycolic acid [103].

### 3.2. Co-Culture in Bone Tissue Regeneration

#### 3.2.1. 2D Co-Culture Models for Bone Regeneration

The usefulness of co-culture systems has been described in the regeneration of many tissues, including bone, nerve, musculoskeletal, and cartilage tissues [2,9,11]. Co-culture systems are frequently investigated in bone regeneration, as more than 2 million bone grafts are performed worldwide each year, making the repair of bone defects a challenge for regenerative medicine [9]. In bone regeneration, two processes (i.e., angiogenesis and osteogenesis) play a crucial role in bone reconstruction [9]. These processes involve different types of cells, and their interactions activate signaling pathways, promote cell-cell junctions, and facilitate cellular stimulation through exosomes and paracrine growth factors [9]. Therefore, many methods were established to incorporate both angiogenic and osteogenic cells in bone repair [9]. Observations showed that successful engraftment of tissue-engineered bone after MSC implantation depends on the rapid establishment of a stable and functional vascular supply [9]. However, prolonged periods of hypoxia and nutrient deprivation will eventually lead to significant cell death during the bone healing process [19]. Therefore, co-culture systems were used to improve the efficiency of bone repair [9]. The co-culture models most frequently applied in bone repair are shown in Figure 2C–E.

Experimental data obtained by Zhao et al. [109] revealed that co-culture of vascular endothelial cells (VECs) and ADMSCs increased the ADMSC osteogenic potential. The authors indicated that the ADMSCs underwent osteogenic differentiation enhanced by VECs in vitro and suggested that the co-culture model of VECs and ADMSCs may be a novel source of cells for bone engineering [109]. Different co-culture set-ups using human monocytes (hMCs) and hMSCs, human osteoblasts with hMCs, or human osteoblasts with osteoclasts were investigated [110]. Direct co-culture systems showed that these systems induced osteoclastogenesis, but active bone resorbing osteoclasts were obtained when M-CSF and RANKL were added to the culture medium [110]. The use of a direct or indirect co-culture system of osteoblasts with osteoclasts increased osteoblastogenesis and decreased osteoclastogenesis and RANKL levels [111]. Ma et al. [112] found that 2D co-culture of hBMSCs and human umbilical vein endothelial cells (HUVECs) showed high osteogenic as well as angiogenic potential. In another study, an indirect co-culture 2D system was used for MSC and endothelial progenitor cell (EPC) co-culture to analyze the osteogenic differentiation of MSCs [113]. MSC/EPC co-culture effectively promoted osteogenic differentiation and enhanced extracellular matrix deposition and mineralization [113]. Another study investigated an indirect 2D co-culture system to define the impact of crosstalk between hDPSCs and hUC-MSCs on osteogenic gene expression as a result of the paracrine effect of both types of cells [114]. The results showed that hUC-MSCs enhanced the osteogenic potential of hDPSCs in co-culture, and this method may be considered in bone tissue engineering [114]. Herath et al. [115] established a triple-cell 2D co-culture model consisting of osteoblasts, endothelial cells, and neutrophils and analyzed the effect of neutrophils on angiogenesis and osteogenesis. The authors observed that the neutrophils induced higher osteogenic marker (ALP, COL1, osteocalcin (OCN), and osteopontin (OPN)) expression in the triple-cell co-culture model compared with the double-cell co-culture model without neutrophils [115]. The angiogenic and osteogenic capacity of rat BMSCs and HUVECs was analyzed in the 2D direct co-culture system under hypoxia conditions [19]. The co-culture system of MSCs and hypoxia-preconditioned HUVECs showed stronger osteogenesis and angiogenesis [19].

#### 3.2.2. 3D Co-Culture Models for Bone Tissue Regeneration

A study by Rong et al. [116] demonstrated that using a co-culture strategy involving hUC-MSCs pre-induced into osteoblasts followed by co-culture with ECs enhanced osteogenesis in vitro and in vivo and facilitated vascularization [116]. It was revealed that osteogenically differentiated hUC-MSCs, cultured in a co-culture system on a 3D-printed tricalcium phosphate (TCP) scaffold, successfully repaired a critically sized calvarial bone defect in rats [116]. The researchers indicated that dual-directional differentiated hUC-MSCs on 3D TCP scaffold improved osteogenesis, and this may be a new approach to fabricating tissue-engineered bioimplants for large bone defect augmentation [116]. Yang et al. [117] induced ADMSCs into osteogenic ADMSCs and endothelial ADMSCs, which they then co-cultured on peptide RADA16-I scaffolds. The results showed that the cells grew well on the scaffolds, and the co-cultured cells exhibited better osteogeneration and vascularization as monolayer culture [117]. The results published by Rozila et al. [118] revealed that co-cultured hADMSCs with human osteoblasts on polycaprolactone (PCL) and hydroxyapatite scaffolds showed the most positive osteogenic differentiation.

A study by Li et al. [119] showed that ECs in indirect co-culture on a hydrogel scaffold promoted proliferation and osteogenic differentiation of osteoblasts through the paracrine signaling pathway. The authors demonstrated that microencapsulated ECs enhanced VEGF secretion, which promoted angiogenesis and enhanced the activation of endothelial cells [119]. The results showed that the microenvironment generated in the 3D indirect co-culture system allowed for the development of tissue engineering strategies that rely strictly on the native bone tissue environment [119]. Tong et al. [120] found that a co-culture system consisting of BMSCs and ECs with adverse conditions such as low serum could significantly reduce apoptosis, increase the proliferation of osteogenic cells, and promote the osteogenic differentiation of BMSCs. HUVECs induced the paracrine effect and promoted the osteogenic differentiation of BMSCs and increased the angiogenic differentiation of HUVECs, thereby enhancing bone regeneration in vivo [120]. Consequently, in vivo studies have confirmed that bone engineering using calcium phosphatase constructs containing both types of cells, namely human periodontal ligament stem cells (hPDLSCs) and human umbilical vein endothelial cells (hUVECs), yields earlier vascularization and more pronounced bone regeneration than using constructs containing only MSCs [121,122]. Borciani et al. [11] summarized the published data on the co-culture systems of osteoblasts and osteoclasts in bone regeneration. In vitro and in vivo experimental studies suggested that co-culture methods could potentially be associated with the advancement of future bone tissue engineering applications [11]. Interesting data have been reported by Maria et al. [111], who showed a high osteogenic effect in the co-culture system using commercially available osteoblasts and donor patient osteoclasts for the therapy of postmenopausal osteopenic women.

In bone tissue regeneration, a novel triple-cell co-culture model consisting of MSCs and ECs with the addition of immunological cells was recently studied [123,124]. The co-culture of MSCs and ECs on hyaluronic acid hydrogels in the presence of M1 macrophages showed good adhesion and proliferation of MSCs and ECs in hydrogel [124]. The authors suggested that macrophages promote the migration of ECs and MSCs into biomaterials and facilitate vascularization. However, this bioconstruction needs further research to confirm its chondro- and osteogenic properties [124]. It is worth mentioning that immunological cells may downregulate the osteogenic potential of MSCs [125]. Tang et al. [125] showed that co-culture of MSCs with monocytes, M1 macrophages, or M2 macrophages on 3D poly (lactic-co-glycolic) acid (PLGA)/PCL scaffolds decreased the osteogenic differentiation of MSCs. Co-cultured monocytes and macrophages decreased the expression of osteogenic markers ALP, bone sialoprotein (BSP), and runt-related transcription factor 2 (RUNX2) [125]. The authors pointed out that the selection of cells for a co-culture system is an important aspect of such approaches [125].

It is worth underlining that bone repair depends not only on cells but also bioactive molecules secreted by cells [9]. Many cytokines and growth factors regulate the crosstalk between endothelial and osteoblastic cells, which are involved in angiogenesis and osteogenesis [9]. It was noted that the co-culture system had an impact on cell communication, and direct co-culture of ECs and MSCs induced paracrine signals that promoted the secretion of VEGF, whereas indirect co-culture of both types of cells induced the paracrine signal, enhancing the secretion of PDGF [109]. The link between the paracrine signaling effect and the co-culture system was observed by other authors [11]. The effect of crosslinking between MSCs and ECs in bone regeneration is presented in Figure 3B.

The TGF-β signaling pathway regulates angiogenesis through the regulation of EC activity, whereas the Notch signaling pathway regulates the expression of vascular endothelial growth factor receptor 1 (VEGFR1) and VEGFR3 and influences the interaction between VEGFs and VEGFRs. Co-culture of ADMSCs and ECs in a 3D collagen gel model promotes VEGFA and VEGFB protein expression, indicating that such interactions modulate angiogenesis in co-culture more significantly than in a monoculture system [126]. Jia et al. [114] found that the Akt/mTOR signaling pathway enhanced crosstalk between hDPSCs and hUC-MSCs in the 3D co-culture system on PCL microspheres and increased both the proliferative activity and expression of osteogenic genes, such as *COL1*, *osteocalcin* (*OCN*), and *osteopontin* (*OPN*) in hDPSCs. For upregulation in 3D co-cultured hDPSCs, the Akt/mTOR signaling pathway might be involved in enhancing cells’ proliferation and osteogenic gene expression [114]. A 3D co-culture system of hDPSCs and ECs was designed using porous microcarriers and applied to bone tissue engineering [127]. The co-cultured construct of hDPSCs and ECs showed significantly higher expression of genes related to osteogenesis (*COL1* and *ALP*) and angiogenesis (*von Willebrand factor* (*vWF*)) as well as *vascular endothelial cadherin* than monocultures [127]. The results indicated that the 3D co-culture cell system can be considered useful as an alternative tool for bone tissue engineering in the future [127]. Interesting data presented by Zhou et al. [128] showed that spheroid co-culturing of BMSCs and osteoblasts resulted in an animal model of a bone-like tissue formation that was similar to the in vivo bone. The implantation of this construct into the tooth extraction sockets of mice allowed it to promote bone formation and acceleration of alveolar bone regeneration [128]. Meanwhile, hDPSC and hPDLSC 3D co-cultures were developed for tooth tissue engineering [129]. Co-culturing of hDPSCs and hPDLSCs on poly-D, L-lactide (PDLLA) improved dental stem cell proliferation and osteogenic differentiation [129].

Another important aspect of bone regeneration is the development of new blood vessels in regenerated tissue [124]. Angiogenesis under human physiological conditions is induced by inflammation. Many inflammatory cells can secrete pro-inflammatory cytokines such as IL-1, IL-6, and IL-8 and pro-angiogenic growth factors (VEGF, bFGF, HGF, and PDGF) to regulate the inflammatory response, which can also influence angiogenesis [123]. It was found that macrophages can secrete tumor necrosis factor-α (TNF-α) and VEGF to promote the migration of ECs and MSCs, which helps facilitate vascularization during bone regeneration [124]. Similarly, neutrophils significantly induce the expression of proangiogenic markers, such as VEGFA, CD34, EGF, and FGF-2 and promote osteogenesis by regulating key osteogenic markers, such as ALP, OCN, OPN, COL1, BMP-2, RUNX2, and ECM proteins [115,124]. With these promising results, more complex co-culture systems may be an advantageous tool for creating prevascular tissue engineering constructs. Co-culture of ADMSCs and ECs in a 3D collagen gel model showed that direct cell-to-cell contact increased the expression of angiogenic genes and proteins, such as VEGFA and VEGFB. The authors suggested that this 3D co-culture model could be useful for cell-based revascularization therapies, including those for bone tissue [126]. Both direct and indirect co-culture of hDPSCs and hPDLSCs on scaffolds increased the proliferation and osteogenic differentiation of these cells [53]. Growing evidence shows that much attention has been given to establishing suitable co-culture conditions to develop effective models and new technological strategies for improving bone repair [127,128,129].

## 4. Conclusions

Cartilage and bone regeneration are still challenging in clinical practice. There are many different questions related to the co-culture system that need to be answered. Numerous research centers have been working on developing a model for cartilage and bone therapy with clinical applications. Currently, various treatment methods are available for cartilage and bone tissue defects caused by trauma or degenerative diseases, including OA. Therapy based on single cells (e.g., chondrocytes, ECs, or MSCs) does not provide stable or long-term recovery of regenerated tissue. It has been observed that, over time, regenerated cartilage often transforms into fibrocartilage, which lacks the mechanical and functional properties of healthy hyaline cartilage. It can be assumed that co-culture systems, especially those on membranes with chondrocytes and MSCs, may dominate future procedures for cartilage regeneration. These systems offer notable advantages over traditional monolayer cell cultures. Firstly, they may closely mimic the natural milieu of cartilage tissue and support the maintenance of the chondrocyte phenotype. Secondly, membranes enable cellular stimulation and interaction not only between the cells themselves but also with the membrane, creating an environment conducive to effective cartilage regeneration. Two mechanisms must be considered in bone regeneration using a co-culture system: angiogenesis and osteogenesis. The presence and interaction between different types of cells induce these processes as in native bone tissue. However, more knowledge is needed on mechanisms induced by interactions between osteogenic and angiogenic cells in a co-culture system, which may lead to positive outcomes in bone regeneration. The results are promising, but there are still too few studies showing a co-culture 2D and 3D system of MSCs and somatic cells, which could help to determine the best models for cartilage and bone regeneration.

## 5. Future Perspectives

For 30 years now, researchers have been working on the best techniques for obtaining hyaline cartilage during cartilage tissue regeneration. In 2001, Quarto et al. [130] presented a milestone in bone regeneration by repairing 4–8 cm bone defects using custom-made porous hydroxyapatite scaffolds covered with autologous BMSCs. Despite this outstanding effort, however, cell-based therapies and tissue engineering are still under scientific pressure to obtain the best biomaterials for cartilage and bone tissue regeneration. It seems that co-culture models may resolve many scientific doubts. A co-culture model allows for the use of cells with different biological properties which may cooperate and, through their crosslink connection with a paracrine effect, enhance the repair of a defect site. However, future studies should focus on the establishment of co-culture conditions, such as the culture medium, time, cell ratio, and appropriate biomaterials for different types of cells for cartilage and bone regeneration.

Further research is needed in order to define the signaling pathways that are important in cell activation in co-culture and establish a model containing proper cells with high chondrogenic potential and showing good osteogenic and angiogenic properties. To date, it is still unclear what model, direct or indirect, is suitable for tissue regeneration. Considerable effort should be put into the biological processes of cells which occur in biomaterials and 3D models to improve tissue engineering.

## Figures and Tables

**Table 1:** Mesenchymal stromal cells frequently used for cartilage and bone tissue engineering [15,34,35,36,37].

Stem Cell Type	Advantages	Disadvantages
**Bone** **Marrow-Derived MSCs (BMSCs)**	- high proliferation rate - strong chondrogenic and osteogenic potential - high chemotactic response to injury - good angiogenic support - clinically well characterized	- invasive harvest procedure - painful clinical procedure - low cell yield (~0.001%) - age-related decline in function - poor survival post-transplantation
**Adipose-Derived MSCs** **(ADMSCs)**	- easy to obtain in large quantities - good osteogenic capacity - strong angiogenic factor secretion (e.g., VEGF or HGF) - low donor site morbidity	- weaker chondrogenic potential than BMSCs - lower ECM production - limited chemotactic migration - donor age impacts on differentiation and proliferation potential
**Human Dental Pulp Stem Cells (hDPSCs)**	- noninvasive collection - good proliferation - high dual chondrogenic and osteogenic potential - enhanced neuro- and angiogenic signaling - promote better angiogenesis than other MSCs	- lower angiogenesis vs. ADMSCs - limited long-term survival - mostly preclinical data
**Human Umbilical Cord MSCs** **(hUC-MSCs)**	- noninvasive collection - potential for allogeneic use (“off the shelf”) - low immunogenicity - high expansion rate - strong angiogenic and immunomodulatory effects	- lower osteogenic potential than BMSCs - less-studied long-term outcomes - still limited in clinical approvals

## Data Availability

No new data were created or analyzed in this study. Data sharing is not applicable to this article.
